# Same species, different diseases: how and why typhoidal and non-typhoidal *Salmonella enterica* serovars differ

**DOI:** 10.3389/fmicb.2014.00391

**Published:** 2014-08-04

**Authors:** Ohad Gal-Mor, Erin C. Boyle, Guntram A. Grassl

**Affiliations:** ^1^The Infectious Diseases Research Laboratory, Sheba Medical CenterTel-Hashomer, Israel; ^2^Bernhard Nocht Institute for Tropical MedicineHamburg, Germany; ^3^Institute for Experimental Medicine, Christian Albrechts University KielKiel, Germany; ^4^Research Center BorstelBorstel, Germany

**Keywords:** *Salmonella enterica*, typhoid, enteric fever, NTS, salmonellosis, gastroenteritis

## Abstract

Human infections by the bacterial pathogen *Salmonella enterica* represent major disease burdens worldwide. This highly ubiquitous species consists of more than 2600 different serovars that can be divided into typhoidal and non-typhoidal *Salmonella* (NTS) serovars. Despite their genetic similarity, these two groups elicit very different diseases and distinct immune responses in humans. Comparative analyses of the genomes of multiple* Salmonella* serovars have begun to explain the basis of the variation in disease manifestations. Recent advances in modeling both enteric fever and intestinal gastroenteritis in mice will facilitate investigation into both the bacterial- and host-mediated mechanisms involved in salmonelloses. Understanding the genetic and molecular mechanisms responsible for differences in disease outcome will augment our understanding of *Salmonella* pathogenesis, host immunity, and the molecular basis of host specificity. This review outlines the differences in epidemiology, clinical manifestations, and the human immune response to typhoidal and NTS infections and summarizes the current thinking on why these differences might exist.

## INTRODUCTION

*Salmonella enterica* is a highly diverse Gram negative bacterial species containing more than 2600 different serovars differentiated by their antigenic presentation. Various serovars are characterized by their host specificity or by the clinical syndrome they cause ranging from asymptomatic carriage to invasive systemic disease. Most *S. enterica* serovars associated with diseases in humans and other warm blooded animals belong to subspecies I consisting of both typhoidal and non-typhoidal serovars. Several excellent recent reviews have highlighted different aspects of invasive salmonellosis (De Jong et al., 2012; Feasey et al., 2012), discussed the mechanisms behind host restriction (Baumler and Fang, 2013), and detailed salmonelloses in immunocompromised individuals (Gordon, 2008; Maclennan, 2014). Here, we will discuss how typhoidal and non-typhoidal serovars differ in epidemiology, clinical manifestations, and the immune response they trigger in humans.

## EPIDEMIOLOGY

While many non-typhoidal *Salmonella* (NTS) serovars such as Typhimurium and Enteritidis are generalist pathogens with broad host specificity, a few *S. enterica* serovars including Typhi, Sendai, and Paratyphi A, B, or C are highly adapted to the human host that is used as their exclusive reservoir. These specialist pathogens, collectively referred to as typhoidal *Salmonella* serovars, are the causative agents of enteric fever (also known as typhoid or paratyphoid fever if caused by serovar Typhi or Paratyphi, respectively). Enteric fever is an invasive, life-threatening, systemic disease with an estimated global annual burden of over 27 million cases, resulting in more than 200,000 deaths (Crump et al., 2004; Buckle et al., 2012). Enteric fever is endemic in the developing world in regions that lack clean water and adequate sanitation, facilitating the spread of these pathogens via the fecal-oral route. In recent years, for unknown reasons, the incidence of infections with serovar Paratyphi A is on the rise and in some regions of the globe, particularly in South–East Asia, this serovar is accountable for up to 50% of all enteric fever cases (Ochiai et al., 2005; Meltzer and Schwartz, 2010).

In contrast to typhoid fever which is common in the developing world, NTS salmonelloses occur worldwide. There are an estimated 93.8 million cases of gastroenteritis due to NTS infection each year, resulting in approximately 155,000 deaths (Majowicz et al., 2010). Despite global morbidity, mortality due to NTS infection is primarily restricted to the developing world. In addition to contaminated animal-derived food products such as poultry, eggs, and dairy products, NTS transmission can result from person to person contact or from contact with pets such as cats, dogs, rodents, reptiles, or amphibians (Hohmann, 2001; Mermin et al., 2004; Braden, 2006; Haeusler and Curtis, 2013). Another important source of infection is consumption of contaminated produce especially sprouts, tomatoes, fruits, peanuts, and spinach which have all been associated with recent outbreaks (Berger et al., 2009, 2010; Barton Behravesh et al., 2011; Cavallaro et al., 2011; Jackson et al., 2013; Bayer et al., 2014).

While normally NTS infections in humans induces gastroenteritis, in up to 5% of NTS cases, bacteria cause an invasive, extra-intestinal disease leading to bacteremia and focal systemic infections, henceforth referred to as invasive NTS (iNTS; Mandal and Brennand, 1988). Interestingly, various NTS serovars (e.g., Typhimurium, Dublin, Choleraesuis, 9,12:l,v:-) tend to have more potential to cause extraintestinal infections than others. This implies there is a genetic basis for the emergence iNTS disease; however, these differences are still not understood (Wilkins and Roberts, 1988; Marzel et al., 2014). In Sub-Saharan Africa, iNTS is a major cause of bacteremia in adults and children, with an estimated annual incidence of 175–388 cases per 100,000 children and 2000–7500 cases per 100,000 HIV-infected adults. Especially *S.* Typhimurium sequence type (ST) 313 is associated with invasive disease. Startlingly, in 20–25% of cases, invasive infection results in the death of the patients. Other major risk factors for invasive disease in addition to HIV are co-infection with malaria and malnutrition (Feasey et al., 2012; Maclennan, 2014).

## CLINICAL MANIFESTATIONS

Enteric fever caused by typhoidal serovars differs dramatically from the gastroenteritis normally associated with NTS. Infections caused by different typhoidal serovars (e.g., Typhi and Paratyphi A) cannot be distinguished by clinical presentation (Meltzer et al., 2005; Patel et al., 2010). The average incubation period for typhoidal serovars is 14 days with symptoms persisting for up to 3 weeks (Olsen et al., 2003; Wangdi et al., 2012). Patients most typically present with a gradual onset of sustained fever (39–40°C). Other frequent symptoms include chills, abdominal pain, hepatosplenomegaly, rash (rose spots), nausea, anorexia, diarrhea or constipation, headache, and a dry cough (Stuart and Pullen, 1946). In contrast to enteric fever, individuals infected with NTS have self-limiting, acute gastroenteritis and watery diarrhea. Nausea, vomiting, abdominal pain, and fever are also common symptoms (McGovern and Slavutin, 1979). With NTS infection, symptoms appear 6–12 h after the ingestion of the pathogen and clinical symptoms last less than 10 days (Glynn and Palmer, 1992). In the case of iNTS infections, which are often associated with patients with immunodeficiency, disease more closely resembles enteric fever in that patients often suffer from high fever, hepatosplenomegaly, and have respiratory complications with intestinal symptoms often being absent.

Both typhoidal and NTS serovars initially adhere to and invade the intestinal epithelium of the small intestine (Liu et al., 1988). Unlike NTS infection, infection by typhoidal serovars does not induce a high inflammatory response during the initial invasion of the intestinal mucosa (Sprinz et al., 1966; Kraus et al., 1999; Nguyen et al., 2004). Minimal intestinal inflammation during enteric fever is correlated with negligible neutrophil transmigration across the intestinal epithelium in contrast to massive neutrophil recruitment during intestinal inflammation caused by NTS serovars (McCormick et al., 1995). In immunocompetent patients, NTS gastroenteritis is self-limiting, with infection being confined to the terminal ileum and colon. In the case of typhoidal salmonellae, after passing the intestinal mucosa, bacteria gain access to underlying lymphoid tissues and multiply intracellularly within mononuclear phagocytes. Infection quickly becomes systemic with spreading of the pathogen from the intestine to the mesenteric lymph nodes, liver, spleen, bone marrow, and gallbladder. Secondary infection of typhoidal organisms to the small bowel can occur via secretion in the bile through the enterohepatic cycle (Gordon, 2008). The absence of robust intestinal inflammation and the lack of neutrophil transmigration are thought to facilitate the invasion of typhoidal serovars into the deeper tissues of the gut and its dissemination to systemic sites (House et al., 2001).

Interestingly, up to 10% of convalescing, untreated patients continue to shed *S.* Typhi in their stool for up to three months after infection (Parry et al., 2002). One to four percent of individuals infected with *S.* Typhi become asymptomatic, chronic carriers that continue to excrete 10^6^–10^10^ *S.* Typhi bacteria per gram of feces for more than 12 months. The role of such chronic carriers in disease transmission was notoriously demonstrated by the case of Mary Mallon (Typhoid Mary). During her work at different households as a cook in the New York City area in the early 20th century, Mary Mallon infected between 26 and 54 people (Marr, 1999). Another example of an asymptomatic *S.* Typhi carrier was “Mr. N” who worked as a cowman and milker in South–East England and was responsible for a 207 case outbreak of typhoid fever, which peaked in 1899 but continued until 1909 (Mortimer, 1999). The suspected site of persistence of *S.* Typhi in carriers is the gallbladder and gallstones are thought to be an important risk factor for developing chronic carriage (Levine et al., 1982) as they are conducive for biofilm formation which protects bacteria from antimicrobial compounds and the host immune system. Long-term carriage of *S.* Paratyphi has received much less attention and is currently less characterized than *S.* Typhi, but a recent study in Nepal suggests a similar rate of persistence for serovars Typhi and Paratyphi A in endemic regions (Khatri et al., 2009; Dongol et al., 2012).

Long-term carriage of NTS has not been described. However, even though symptoms usually last only for a few days, adults excrete *Salmonella* on average for 1 month after infection and children under the age of 5 years shed bacteria in their feces for an average of 7 weeks (Buchwald and Blaser, 1984; Hohmann, 2001). Interestingly, several studies have shown that treatment with antibiotics can prolong shedding of NTS bacteria (Aserkoff and Bennett, 1969; Murase et al., 2000), although these findings are controversial (Dryden et al., 1996; Hohmann, 2001). In comparison to NTS serovars, the long-term persistence of typhoidal serovars in humans suggests an enhanced ability of these pathogens to evade the human immune system (Raffatellu et al., 2008b).

## HUMAN IMMUNE RESPONSE

Infection in humans by NTS serovars induces a strong Th1 response with high levels of IFN-γ, IL-18, IL-12, IL-15, TNF-α, and IL-10 detected in serum from patients (Mizuno et al., 2003; Stoycheva and Murdjeva, 2005). Expression of several chemokines is also induced upon NTS infection, which leads to the recruitment and activation of macrophages and dendritic cells, and a significant influx of neutrophils into the intestinal lumen, which is a hallmark of NTS gastroenteritis. The fact that typhoidal serovars are not typically associated with acute diarrhea or a strong influx of neutrophils into the intestinal lumen (Sprinz et al., 1966; Kraus et al., 1999; Nguyen et al., 2004) suggests that their initial interaction with the human gut mucosa is less inflammatory than that of NTS serovars.

Recent studies have shown that patients with inherited deficiency of the IL-12/IL-23 system (IL-12p40/IL-12Rβ1) are highly susceptible to NTS infections, but not to *S.* Typhi or *S.* Paratyphi infections, even though some of these patients live in endemic areas (MacLennan et al., 2004; Van de Vosse and Ottenhoff, 2006). These observations support the possibility that different inflammatory pathways may be involved in NTS vs. typhoidal infections including a distinct role for the IL-12 pathway. This idea is further supported by additional epidemiological observations indicating that invasive infections caused by NTS, but not by typhoidal serovars, are often associated with immunocompromised adults, in particular individuals infected with HIV (Gordon, 2008; MacLennan and Levine, 2013). This implies that certain immune responses, malfunctioning during HIV infection, are required for the immune defense against systemic infection of NTS, but not against typhoidal serovars.

The immune response to infection with typhoidal serovars is complex and involves both humoral and cell-mediated immune responses (Sztein, 2007). Clinical studies that examined the immune response of patients infected with *S.* Typhi showed a significant CD4 and CD8 T cell response to specific *S.* Typhi antigens during typhoid fever, with elevated levels of IFN-γ during the acute phase of the disease (Butler et al., 1993; Sheikh et al., 2011). Transcriptome analysis of peripheral blood mononuclear cells (PBMCs) from patients with acute typhoid fever also demonstrated up-regulation of the genes from the IFN-γ pathway compared to healthy individuals (Thompson et al., 2009). Induction of other cytokines in response to *S.* Typhi infection include IL-6 and IL-8 which are secreted into the serum during the acute phase of typhoid fever (Butler et al., 1993; Keuter et al., 1994; Gasem et al., 2003). PBMCs from immunized volunteers orally vaccinated with an attenuated *S.* Typhi vaccine secrete Th1 cytokines including IFN-γ, TNF-α, and IL-10, following sensitization with a number of *S.* Typhi antigens including flagella (Wahid et al., 2007). Collectively, these findings indicate that the human immune response to *S.* Typhi infection is predominantly Th1-associated.

Given that typhoidal serovars do not typically illicit septic shock, in contrast to many other Gram-negative pathogens that induce bacteremia and leukopenia (Pohan, 2004; Tsolis et al., 2008; Gal-Mor et al., 2012), suggests a restrained immune response mediated by these pathogens in the human host. This view is consistent with the clinical observation that serum levels of pyrogenic cytokines IL-1β and TNF-α are relatively low in patients with typhoid fever compared to the levels found in patients with sepsis caused by other Gram-negative pathogens. In fact, IL-1β and TNF-α production by PBMCs has been shown to be suppressed during the acute phase of typhoid fever (Butler et al., 1978; Girardin et al., 1988; Keuter et al., 1994; Gasem et al., 2003).

Despite the increasing prevalence of *S.* Paratyphi A in endemic regions, the immune response to *S.* Paratyphi infection is much less characterized than the one to *S.* Typhi. A recent study done in our group examined the circulating cytokine profile of healthy Israeli travelers that became infected with *S.* Paratyphi A during an outbreak in Nepal. Comparison of 16 cytokines demonstrated considerable (more than 10-fold) increase in the serum concentration of IFN-γ, but only a moderate elevation in the concentration of IL-6, IL-8, IL-10, and TNF-α between convalescence and the peak time of clinical presentation (Gal-Mor et al., 2012). These results suggest that the prominent IFN-γ and the moderate TNF-α, IL-6, and IL-8 responses are common to both typhoid and paratyphoid fever. Interestingly, no changes in IL-12 serum concentrations were detected during the acute phase of the disease (Gal-Mor et al., 2012), in contrast to its induction seen during gastroenteritis caused by NTS serovars (Stoycheva and Murdjeva, 2005).

## CURRENT THERAPIES AND VACCINES

Antibiotic therapy can prolong the duration of excretion of NTS and therefore is only recommended for people with severe illness, invasive disease, or for certain risk groups including infants, the elderly, and immunocompromised individuals. Enteric fever, on the other hand is always immediately treated with antibiotics. In the 1990s, physicians moved away the first-line antibiotics chloramphenicol, ampicillin, and cotrimoxazole due to widespread resistance amongst *S. enterica* serovars. Since then, fluoroquinolones (like ciprofloxacin) have been the primary treatment for salmonelloses, as this class of drug is particularly effective against intracellular Gram-negative bacteria. While there is increasing resistance to fluoroquinolones, new fluoroquinolones like gatifloxacin hold promise. Third generation cephalosporins are often the second-line treatment to treat salmonelloses. In addition, azithromycin is relatively new drug with activity against both nalidixic acid resistant and multidrug resistant (MDR) strains (Hohmann, 2001; Arjyal and Pandit, 2008).

Multidrug-resistance is an increasing problem in *S. enterica* serovars. Resistance to multiple antibiotics is especially common in serovars Typhimurium and Newport and multidrug-resistant strains are also linked to more severe disease outcome (Krueger et al., 2014). Notably, many strains of *S.* Typhimurium Definitive Type (DT) 104, which have caused multiple outbreaks since the 1990s, are resistant to ampicillin, chloramphenicol, streptomycin, sulphonamides, and tetracycline (Mather et al., 2013). Moreover, new resistant strains of *S. enterica* are continuously emerging worldwide. For example, an MDR strain of serovar Infantis now accounts for up to 35% of the NTS infections in Israel (Gal-Mor et al., 2010; Aviv et al., 2014). Additional examples are the emergence of resistant strains of serovars Virchow (Weill et al., 2004) and Heidelberg (Dutil et al., 2010). Similarly, many iNTS strains are resistant against ampicillin, chloramphenicol, kanamycin, streptomycin, trimethoprim, and cotrimoxazole (Gordon, 2008; Kingsley et al., 2009; Msefula et al., 2012). Therefore, there is a high need to (i) prevent further resistance development through the prudent use of antibiotics, (ii) improve measures that prevent spread of MDR strains, and (iii) discover new therapies for salmonelloses. Interestingly, the re-emergence of chloramphenicol sensitive strains in areas where resistance was previously prevalent suggests that cycling or rotation of antibiotics could also be an effective strategy to deal with antibiotic resistance, rendering older antibiotics useful once again (Abel Zur Wiesch et al., 2014).

Three types of vaccines against *S.* Typhi are currently commercially available, but unfortunately, there is still not a single licensed vaccine available against *S.* Paratyphi A, with very little, if any, cross-protection provided by the available *S.* Typhi vaccines. Vaccination strategies against typhoid fever including a description of ongoing trials were recently reviewed in detail (Waddington et al., 2014). The currently licensed *S.* Typhi vaccines include (i) a killed whole cell parenteral vaccine (Engels et al., 1998), (ii) a live attenuated oral vaccine, designated Ty21a (Germanier and Fuer, 1975) and, (iii) a Vi polysaccharide capsule-based vaccine (Tacket et al., 1986). There are vaccines against NTS serovars Enteritidis and Typhimurium which are effective in poultry (Desin et al., 2013). However, there are no vaccines available for NTS in humans or other animal reservoirs such as cattle or pigs. This represents a significant limitation in the existing prevention strategies. Understanding the host specificity determinants of *S. enterica* serovars will aid in future therapeutic and vaccine development.

## WHY DO TYPHOIDAL AND NTS SEROVARS ELICIT SUCH DIFFERENT HOST IMMUNE RESPONSES?

How do pathogens so similar, belonging to the same subspecies (*S. enterica* ssp. I), with >96% DNA sequence identity between shared genes (McClelland et al., 2001) induce such different clinical manifestations and immune responses in humans? Despite significant advances in the field, this question is still far from being answered. Understanding the genetic and molecular mechanisms responsible for differences in disease outcome will aid in our understanding of *Salmonella* pathogenesis, host immunity, and the molecular basis of host specificity (**Table 1**).

**Table 1:** Summary of the differences between NTS and typhoidal serovars associated with disease in humans.

	NTS serovars	Typhoidal serovars
Serovars	Represented by the ubiquitous serovars Typhimurium and Enteritidis, but ∼1500 other serovars of *S. enterica* ssp. I are known	Typhi, Paratyphi, and Sendai
Host range	Broad	Human-restricted
Epidemiology	Worldwide	Endemic in developing countries especially Southeast Asia, Africa, and South America
Reservoirs	Farm animals, produce, pets	None, human to human transmission
Clinical manifestations	Self-limiting gastroenteritis in immunocompetent individuals (diarrhea, vomiting, cramps)In immunocompromised patients (including patients with inherited deficiency of the IL-12/IL-23 system and HIV), disease is associated with invasive extraintestinal infections	Invasive, systemic disease in immunocompetent individuals (fever, chills, abdominal pain, rash, nausea, anorexia, hepatosplenomegaly, diarrhea or constipation, headache, dry cough)
Disease course	Short incubation period (6–24 h) Brief duration of symptoms (less than 10 days) Long-term carriage has not been observed	Long incubation period (7–21 days) Extended duration of symptoms (up to 3 weeks) One to four percent of infected individuals become long-term (≥1 year) carriers
Human immune response	Robust intestinal inflammation, neutrophil recruitment, Th1 response	Minimal intestinal inflammation, leukopenia, Th1 response
Genetic basis of disease differences and host specificity	Low degree of genome degradation Able to use terminal electron acceptors for anaerobic respiration in the inflamed gut Unique virulence factors (e.g., fimbriae, SPI-14)	∼5% of the genome is degraded (e.g., inactivated metabolic and virulence factor genes) Unique virulence factors and pathogenicity islands (e.g., Vi antigen, SPIs 7, 15, 17, and 18)
Vaccination	No vaccine available for humans	(i) killed whole cell parenteral vaccine, (ii) live attenuated oral vaccine (Ty21a), (iii) Vi polysaccharide capsule-based vaccine
Animal models of human disease	Streptomycin-pretreated mice Calves Non-human primates	Mouse infection with *S.* Typhimurium *Tlr11^-/-^* mice Humanized mice

**FIGURE 1:**
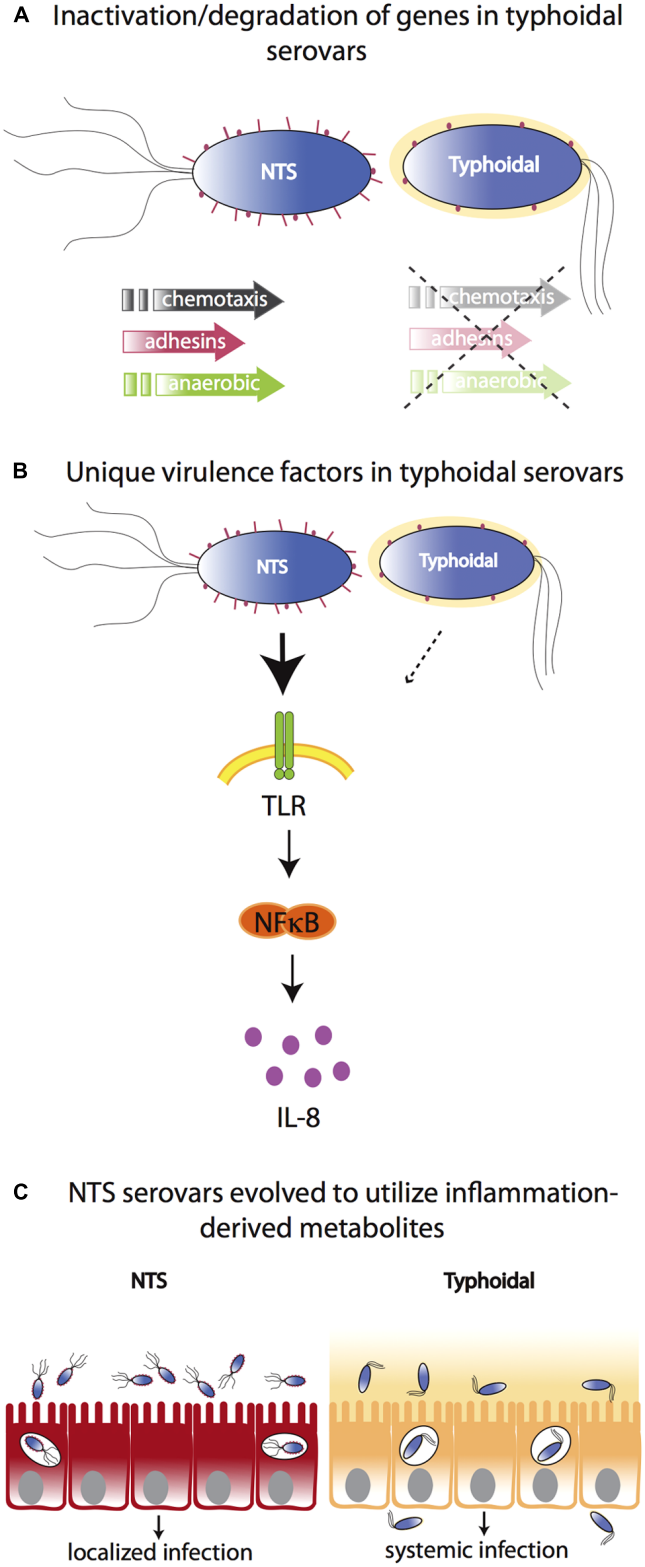
**Molecular bases for differences between typhoidal and NTS serovars. (A)** Typhoidal serovars possess several inactive/degraded genes compared to NTS serovars such as genes for chemotaxis, adhesion, and anaerobic metabolism. **(B)** Both typhoidal and NTS serovars possess unique virulence factors. For example, some *S.* Typhi strains express Vi capsule that reduces TLR-dependent IL-8 production in the intestinal mucosa. However, while the Vi capsule plays a role in typhoid fever manifestation, it is not necessary as it is absent from other typhoidal serovars and Vi-negative mutants of *S.* Typhi are still able to cause a typhoid-like illness in humans. **(C)** In contrast to typhoidal serovars, NTS cause severe intestinal inflammation. NTS serovars have evolved to utilize inflammation-derived metabolites (e.g., nitrate and tetrathionate), thereby enhancing their growth in the inflamed intestine. Typhoidal serovars have lost the ability to benefit from inflammation-derived metabolites and disseminate to systemic sites to a much greater extent.

*In vitro* tissue culture studies suggest that *S.* Typhi induces restrained inflammatory responses that do not trigger a pro-inflammatory response via TLR5. Similarly, polarized human colonic epithelial (T84) cells infected with *S.* Typhi induce significantly lower levels of the neutrophil chemoattractant IL-8 compared to *S.* Typhimurium infection (Raffatellu et al., 2005). Raffatellu et al. (2008b) have therefore postulated that *S.* Typhi expresses unique virulence factors that allow this pathogen to overcome the innate immune response in the intestinal mucosa resulting in the absence of neutrophil infiltration and inflammatory diarrhea. One of the current hypotheses in the field suggests that the polysaccharide capsular antigen Vi in *S.* Typhi enables this pathogen to resist phagocytosis and complement killing (Robbins and Robbins, 1984) and masks access to pattern recognition molecules, resulting in less IL-8 production (Raffatellu et al., 2005), limited neutrophil influx, and thereby reduced small bowel inflammation (Sharma and Qadri, 2004; Wilson et al., 2008). The role of the Vi antigen regulator TviA, and its putative contribution to *S.* Typhi’s ability to evade the immune system have been recently reviewed (Wangdi et al., 2012). Nevertheless, since the Vi capsule is largely restricted to serovar Typhi and is absent from serovars Paratyphi A and Sendai, it cannot explain why the clinical manifestations of these other typhoidal serovars differ from that of NTS. Furthermore, the fact that Vi-negative mutants of *S.* Typhi are still able to cause a typhoid-like illness in human volunteers (Zhang et al., 2008), suggests that additional mechanisms are involved (**Figure 1**).

Of the approximately 4400 *S.* Typhi and *S.* Paratyphi A genes, about 200 are inactivated or functionally disrupted, while most of their homologs in *S.* Typhimurium are intact. Many of the degraded genes found in the genomes of the typhoid serovars are involved in motility and chemotaxis or encode for type 3 secretion system effectors, fimbriae, or adhesins that play a role in *Salmonella* pathogenicity (McClelland et al., 2004). Furthermore, *Salmonella* pathogenicity island (SPI)-7 (encoding the Vi antigen), SPI-15, SPI-17, and SPI-18 are present in the genome of *S.* Typhi, but not in the genome of *S.* Typhimurium, while SPI-14, present in *S.* Typhimurium, is absent from the genome of typhoidal serovars (Sabbagh et al., 2010). Therefore, it is highly possible that differences in virulence and colonization factor composition affect host–pathogen interactions and disease outcome in humans. This notion has recently been demonstrated by the expression of the *S.* Typhimurium effector, GtgE, in *S.* Typhi. When secreted into host cells, GtgE proteolytically degrades Rab29 and confers the ability of *S.* Typhi to survive and replicate within macrophages and in tissues from mice, a normally non-permissive host (Spano and Galan, 2012).

Recent evidence suggests that NTS serovars have evolved to flourish in the inflamed gut environment and use inflammation to outcompete microbiota (Stecher et al., 2007; Thiennimitr et al., 2011). It has been proposed that typhoidal strains may have lost this ability and therefore have evolved to not induce inflammation in the gut but rather thrive systemically. For example, a by-product of the acute intestinal inflammation triggered by *S.* Typhimurium and other NTS serovars is the generation of the terminal electron acceptors nitrate and tetrathionate in the lumen of the inflamed gut. These compounds can be used by *S.* Typhimurium and other NTS serovars to outcompete the fermenting gut microbes that are unable to utilize these electron acceptors (Winter et al., 2010). In another recent report, Nuccio and Baumler (2014) have identified a network of 469 genes involved in central anaerobic metabolic pathways that are intact in NTS, but are decayed in the genome of typhoid serovars. Some of these degraded genes include the ethanolamine utilization pathway (*eut* genes) as well as the vitamin B_12_ biosynthesis pathway (*cbi* and *cob* genes) required for ethanolamine utilization (Nuccio and Baumler, 2014). These pathways are hypothesized to enable NTS to utilize inflammation-derived nutrients to outcompete other gut microbes.

Collectively, a substantial degree of metabolic and virulence gene degradation exists in the genomes of typhoidal serovars which may explain the restricted host-tropism of these pathogens and may also provide at least a partial explanation as to why typhoidal and NTS-infections induce such different clinical presentations and immune responses in humans.

## ANIMAL MODELS

### ANIMAL MODELS OF NON-TYPHOIDAL SALMONELLOSES

There are several animal models used to model human gastroenteritis caused by NTS. The model which most resembles human disease is arguably infection of non-human primates (Kent et al., 1966; Rout et al., 1974). Rhesus macacques are especially useful for investigating co-infection with simian immunodeficiency virus. For example, infection of SIV-infected macacques with *S.* Typhimurium results in a blunted immune response and invasive bacterial disease similar to what is seen in HIV-infected patients (Raffatellu et al., 2008a). Furthermore, this model is useful for testing the efficacy and safety of potential live *Salmonella* vaccines for HIV infected people (Ault et al., 2013). However, the use of primates is limited by ethical concerns, cost, and the inability for genetic manipulation. Infection of calves with *S.* Typhimurium results in similar pathology to humans. Furthermore, *S.* Typhimurium is a natural pathogen of cattle and beef is a common reservoir for human infection (Santos et al., 2001; Costa et al., 2012). Data from the calf model have provided valuable insights into host–*Salmonella* interaction. However, this model is also restricted by cost and the limited possibility for genetic manipulation of the host.

Due to the low cost, ease of housing/handling, and genetic manipulation possible, mouse models are the most widely used animal models to study bacterial disease. NTS infection of mice does not mimic gastroenteritis as seen in humans but results in a typhoid-like systemic disease. However, after pretreatment of mice with antibiotics such as streptomycin or kanamycin, *S.* Typhimurium can overcome the “colonization resistance” presented by the natural microbiota and thus efficiently colonize the cecum and colon. In the now widely used streptomycin pretreatment model, NTS infection has been shown to lead to overt inflammation characterized by transmural inflammation including epithelial destruction, infiltration of inflammatory cells into the mucosa, formation of crypt abscesses, submucosal edema, and hyperplasia (Barthel et al., 2003; Hapfelmeier and Hardt, 2005; Sekirov et al., 2008; Woo et al., 2008). This model is now being exploited by many research groups to dissect both the bacterial- and host-mediated mechanisms involved in intestinal inflammation induction by NTS.

### ANIMAL MODELS OF ENTERIC FEVER

*S.* Typhi, *S.* Paratyphi, and *S.* Sendai are human-restricted pathogens. Historically, attempts at eliciting enteric fever in animal models by infection with *S.* Typhi have proven to be rather inadequate. Chimpanzees infected with *S.* Typhi develop a mild disease that resembles enteric fever, but only when infected with a very high dose of 1 × 10^11^ CFU (Edsall et al., 1960). Another model for *S.* Typhi consists of inoculating mice intraperitoneally with *S.* Typhi suspended in hog gastric mucin (Pasetti et al., 2003). However, this model has not been found to correlate well with human enteric fever and with the expected attenuation of key *Salmonella* virulence regulators, such as PhoP (Baker et al., 1997).

Therefore until recently, due to the lack of suitable animal models, much of our understanding of enteric fever had been extrapolated from *S.* Typhimurium infection in mice. Mice infected with *S.* Typhimurium display minimal intestinal pathology but become systemically colonized as seen in humans with enteric fever. This model also allows for investigation of gallbladder colonization which is most likely the niche for chronic *S.* Typhi carriage in humans (Menendez et al., 2009; Gonzalez-Escobedo et al., 2013). Susceptible (*Slc11a1*^-/-^, also known as *Nramp1*) mouse strains have been widely used but also resistant (*Slc11a1*^+/+^) mice have proven useful. Mice with a wild-type *Slc11a1* gene (e.g., 129Sv, DBA) are relatively resistant to high doses of *S.* Typhimurium and have been particularly useful to investigate chronic infection, carriage (Lawley et al., 2006; Monack et al., 2004), and transmission (Lawley et al., 2008; Gopinath et al., 2012; Monack, 2012). In general, infection of mice with NTS has provided invaluable insight into the role of specific virulence factors in host invasion, dissemination, and transmission and although the murine inflammatory response to NTS in some ways resembles the human response to typhoidal serovars (Santos et al., 2001), conclusions from this model regarding the relevance to human typhoid disease must be carefully inferred.

In recent years, more sophisticated mouse models have been developed to study *S.* Typhi infection. Mathur et al. (2012) have shown that *Salmonella* flagellin is recognized in the mouse intestine by Toll-like receptor 11 (TLR11), which is absent from humans. *Tlr11* knockout mice are severely attenuated in innate epithelial responses to *S.* Typhi (and *S.* Typhimurium) and exhibit significant systemic infection following oral administration (Mathur et al., 2012; Shi et al., 2012). It will be exciting to see if this model can also be used for infection with *S.* Paratyphi.

Another promising novel model is the use of humanized mice whereby immunodeficient mice (either *Rag2^-/-^ Il2rg^-/-^* or NOD⋅Cg-*Prkdc^scid^ Il2rg^-/-^*) lacking murine T, B, and NK cells are engrafted with human CD34+ hematopoietic stem cells (Shultz et al., 2007). These chimeric mice contain human immune cells including B cells, CD4^+^ and CD8^+^ T cells, NK cells, monocytes, and myeloid and plasmacytoid dendritic cells. Such humanized mice facilitate *S.* Typhi replication in the liver, spleen, and gallbladder and allow long-term persistence to be modeled (Song et al., 2010; Firoz Mian et al., 2011). In addition, infection results in a progressive, lethal infection within two to three days with inflammatory cytokine responses resembling human typhoid (Libby et al., 2010). These models suggest that the presence of human immune cells is prerequisite for systemic infection and *in vivo* replication of *S.* Typhi in the mouse. Although these humanized mice have proven informative to the study of *S.* Typhi infection, they are expensive and labor-intensive models and (so far) not widely used. Another limitation of such models is that they are subject to considerable inconsistency as a result of the genetic heterogeneity of donors and the variable degree of engraftment (Libby et al., 2010; Mian et al., 2011).

## PERSPECTIVES

In-depth comparative analyses of the genomes of *Salmonella* serovars have begun to explain the basis for the variation seen in disease manifestations; however, this is still far from being fully understood. An interesting question in this regard is whether there is a genetic basis for the emergence of iNTS strains and why some NTS serovars (e.g., Typhimurium, Dublin, Choleraesuis, Schwarzengrund) tend to cause more invasive disease than others. In addition, the mechanisms by which co-infections (e.g., with *Plasmodium falciparum*, HIV) contribute to the increased risk of iNTS bacteremia must be further investigated. From the perspective of the host response, one unanswered question is whether there are unique immune responses to different typhoidal strains (e.g., Typhi vs. Paratyphi). And lastly, a fast-developing area of research that has already had implications on our understanding of salmonelloses is that of the role of the microbiota in disease outcome (see review by Santos in this issue). In the case of gastrointestinal pathogens, the influence of the host microbiota on pathogenesis, host immunity, and disease progression can no longer be overlooked.

Exploitation of the recent advances in modeling typhoid and NTS infection in mice is likely to provide novel insights into how these serovars are able to cause such different diseases. Opportunities remain, however, in the development of “next generation” humanized mouse models with enhanced human cell engraftment and function. These models hold much promise as they allow one to study the pathogenesis of human-restricted serovars, as well as to test the efficacy of therapeutic agents and experimental vaccines. Understanding the genetic and molecular mechanisms responsible for differences in disease outcome will aid in our understanding of *Salmonella* pathogenesis, host immunity, and the molecular basis of host specificity. Together, this information may be applied to control *Salmonella* infection, with specific determinants being targeted for therapeutic and vaccine development.

## Conflict of Interest Statement

The authors declare that the research was conducted in the absence of any commercial or financial relationships that could be construed as a potential conflict of interest.
